# Engineering of Nanofibrous Amorphous and Crystalline Solid Dispersions for Oral Drug Delivery

**DOI:** 10.3390/pharmaceutics11010007

**Published:** 2018-12-24

**Authors:** Laura Modica de Mohac, Alison Veronica Keating, Maria de Fátima Pina, Bahijja Tolulope Raimi-Abraham

**Affiliations:** 1 DIBIMIS Department, University of Study of Palermo, 90128 Palermo, Italy; laura.modicademohac@unipa.it; 2 Drug Delivery Group, Institute of Pharmaceutical Science Faculty of Life Sciences and Medicine, King’s College London, London SE1 9NH, UK; Alison.Keating@kcl.ac.uk; 3 Department of Pharmaceutics, University College London School of Pharmacy, London WC1N 1AX, UK; mariafatima.gpina@gmail.com

**Keywords:** solid dispersion, aqueous solubility enhancement, amorphous, crystalline, oral drug delivery, electrospinning

## Abstract

Poor aqueous solubility (<0.1 mg/mL) affects a significant number of drugs currently on the market or under development. Several formulation strategies including salt formation, particle size reduction, and solid dispersion approaches have been employed with varied success. In this review, we focus primarily on the emerging trends in the generation of amorphous and micro/nano-crystalline solid dispersions using electrospinning to improve the dissolution rate and in turn the bioavailability of poorly water-soluble drugs. Electrospinning is a simple but versatile process that utilizes electrostatic forces to generate polymeric fibers and has been used for over 100 years to generate synthetic fibers. We discuss the various electrospinning studies and spinneret types that have been used to generate amorphous and crystalline solid dispersions.

## 1. Introduction

Approximately 40% of marketed oral drugs are categorized as having poor aqueous solubility (i.e., <0.1 mg/mL) [1] resulting in a significant challenge in drug development. Several formulation strategies such as co-crystals [2], salt formation [3], particle size reduction [4], and more commonly, solid dispersion (SD) approaches [5,6,7] have been employed to increase aqueous solubility (and in turn enhance the oral bioavailability) of Biopharmaceutics Classification System (BCS) II (i.e., low solubility and high permeability) and IV (i.e., low solubility and permeability) drugs.

SDs have been redefined several times over time. For example, in 1961, Sekiguchi and Obi suggested that when the drug was formulated as a SD, it was present in a microcrystalline state as a eutectic mixture i.e., a combination of crystalline components (drug and a carrier) that are miscible in the molten liquid state, but show little to no miscibility in the solid state and on cooling would crystallize as two components [8]. Later, Chiou and Riegelman (1971) referred to SDs as a dispersion of one or more active ingredients present within an inert carrier or matrix in the solid state prepared by the melting (fusion), solvent-based, or melting-solvent method [9]. This was further defined by Lerner et al. (2000) as a dispersion in which the drug is only partially molecularly dispersed and is generated from physical mixtures of the carrier and drug [10]. In 2002, Craig described SDs as a delivery system whereby the drug is dispersed in a biologically inert matrix, to enhance oral bioavailability [11]. A range of materials are used as the inert matrix or carrier in a SD ranging from sugars [12,13], wax based systems [14] and, more commonly, polymers.

**Figure 1:**
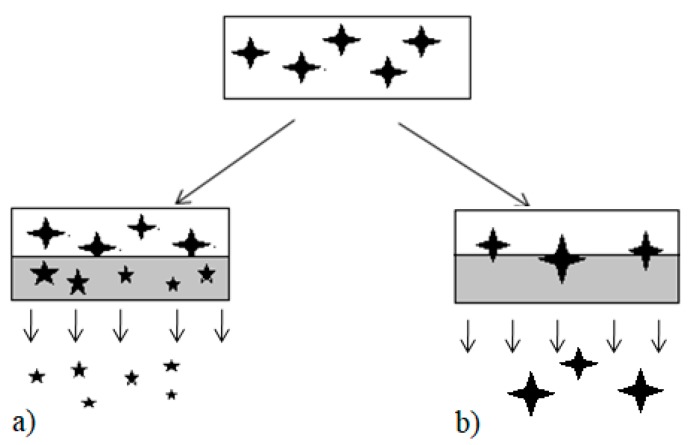
Schematic diagram shows (**a**) Carrier-controlled dissolution and (**b**) drug-controlled dissolution (Adapted with permission from [11], copyright Elsevier, 2002). *The larger 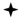 represents dissolved drug, the smaller 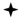 represent the partially dissolved drug. Grey box represents an aqueous environment.*

There are several hypotheses regarding the mechanism of drug release from SD systems amorphous and crystalline alike. The most agreed hypothesis is the existence of two different mechanisms of drug release, carrier-controlled or drug-controlled (Figure 1). This hypothesis is based on key findings by Corrigan et al. [15] in their drug release study of a phenobarbitone-(poly)ethylene glycol (PEG) SD where the rate of phenobarbitone release from the SD was the same as that of PEG on its own. Craig [11] further suggested that, if a system underwentcarrier-controlled dissolution then the physical properties such as initial particle size of the drug or crystallinity should be largely irrelevant. In comparison with drug-controlled dissolution, the physical property of the drug as well as its solubility is of greater relevance [11], highlighting the potential of SDs to enhance drug dissolution rate through a preferential selection of the appropriate carrier (in this case polymer) or a combination of carrier and carrier types (e.g., wax and polymers polymer blends [14]).

In most cases where SDs are generated, a comparison is made with the physical mixture (PM). The PM is essentially where the drug and carrier are not chemically combined through a process but prepared through gentle or mild mixing. To ensure drug release compared to SD is not influenced by additional factors such as particle size, it is good practice for both drug and carrier to be passed through a dry sieve of known aperture so any effects observed are due entirely to the properties of the PM. Examples in the literature exist that demonstrate enhanced drug release of a PM compared to its SD counterpart. For example Leonardi and Salomon (2013) PM of benznidazole and PEG 6000 offered greater dissolution compared to it a SD prepared by a solvent method [16].

SDs have been characterized into two main classifications, firstly in accordance to carrier type and secondly taking into account the correlation to stability and solubility, preparation, and characterization techniques [17].

When classifying SDs by carrier type, there are four generations that have been discussed and explored in the literature. The first generation SDs were introduced by Sekiguchi and Obi as eutectic mixtures where both drug and carrier were present in the crystalline state [18]. Second generation SDs were carriers are generally polymeric and present in the amorphous state with the drug dispersed molecularly within the inert matrix. The drug is generally supersaturated to ensure its solubilization in the carriers in second generation SDs [19]. Third generation SDs were the carriers can be a co-polymer system or a mixture of surfactants and polymers and improvement of the drug release profile is due to the carrier’s surface activity or self-emulsifying properties which in addition could reduce drug re-crystallization and improve SD stability [20]. Finally, fourth generation SDs are the carriers are bifunctional in nature and have solubilizing as well as surfactant properties [21]. The carriers are non-ionic and so the solubility is independent of pH changes making these carriers more suited for SD development. Soluplus^®^, a polyvinyl caprolactam-polyvinyl acetate-polyethylene glycol graft co-polymer solubilizer with an amphiphilic chemical structure has been highlighted as a fourth generation SD carrier [22]. 

When classifying SDs in accordance to their correlation to stability and solubility, preparation and characterization techniques, six classes have been identified [23]. Class C–C and Class C–A where the Active Pharmaceutical Ingredient (API) is present in the crystalline state are dispersed in either a crystalline or amorphous carrier respectively. Class C–C and C–A SDs are deemed stable, however, less soluble due to the crystalline nature of the API. Class A–C and Class A-A were the API is present in the amorphous state dispersed in either a crystalline or amorphous carrier. Class A–C and CC SDs are considered to increase API solubility enhancement, however, low miscibility is observed between the API and carrier has been highlighted as an issue. Class M–C and Class M–A are where the drug is molecularly dispersed within either a crystalline or amorphous carrier. These systems have been shown to greatly improve drug dissolution behavior with great physical stability due to the high miscibility and strong molecular interaction between the two components with the API being molecularly dispersed [23].

In this review, we discuss amorphous and crystalline SDs which we define as a system where the drug is molecularly dispersed with an amorphous polymer matrix and as a system where the micronized or nanosized crystalline drug is dispersed within an amorphous polymer matrix with or without the use of stabilizing agents respectively.

SD stability has been studied at length over the years and factors influencing can be summarized as follows: physical stability, crystallization tendency, amorphization, drug-polymer miscibility, dissolution enhancement, and method of preparation. Electrospinning is a rapid solvent evaporation method and is well suited for drugs with thermal instability and can avoid drug re-crystallization or amorphization during processing [24]. Many polymers have been used to prepare SDs such as poly(*N*-vinvylpyrrolidone) (PVP) [25], polyethylene glycol (PEG) [26], zein [27], hydroxypropyl methylcellulose (HPMC) [28,29], polycaprolactone [30], and polyacrylic acid [31], expanding the potential of processes used to make SDs from the traditional solvent-based methods [32,33], hot melt extrusion (HME) [34,35,36], spray-drying [22,37], to polymer-based nanofabrication methods such as electrospinning [38,39,40] and other centrifugal spinning based methods [12,13,41,42].

In this review, we focus primarily on the emerging trends in the generation of amorphous and crystalline SDs using nanofabrication techniques namely electrospinning to improve the dissolution rate and in turn bioavailability of poorly water-soluble drugs. It is important to note that there are more studies investigating the generation of electrospun nanofibrous amorphous solid dispersions than crystalline SDs suggesting that electrospun nanofibrous crystalline SDs are still a relatively under-investigated formulation strategy.

## 2. Nanofibrous Amorphous Solid Dispersions

Amorphous materials have higher enthalpy, entropy, and free energy compared to their crystalline counterparts, leading to higher apparent solubility. The weaker attractive intermolecular forces of amorphous materials are more easily broken, allowing molecules to move from the material’s surface into the medium greater ease compared to crystalline counterparts. As a result, amorphous materials are more soluble and have a faster dissolution rate. Unfortunately, even with such advantages the success and usefulness of amorphous materials in drug development is greatly dependent on their long stability within a dosage form [3,43,44]. Given the high solubility of amorphous compounds (compared to their crystalline counterparts), amorphous solid dispersions (ASDs) are an important strategy to improve the apparent solubility, dissolution rate, and in turn bioavailability of poorly water-soluble drugs [45].

Nanofibers possess a high surface area to volume ratio and have gained increased interest in drug delivery beyond their known application in areas such as tissue engineering scaffolds [46] or wound dressing for example [47]. Due to their high drug-loading capacity, internal architecture, porosity, and malleability the addition of therapeutically relevant molecules has several benefits. For further information on recent developments in the application of micro and nanofabrication techniques in drug delivery and on nanofibres in drug delivery, the reader is directed to a recent review by Qi and Craig [26].

### 2.1. Electrospinning

Electrospinning is a simple but versatile process that utilizes electrostatic forces to generate polymeric fibers and has been used for over 100 years to generate synthetic fibers [48,49,50]. Typically, electrospinning involves pushing a viscous polymer or polymer/drug solution through a spinneret (narrow gauge syringe needle) at a constant flow rate. A voltage is applied to the polymer solution, creating repulsive forces between the like charges in the solution and attractive forces between the charged solution and the grounded collector. When the electrostatic forces equal the surface tension of the liquid, a Taylor cone is formed. If the electric field is increased beyond this point, the electrostatic repulsion will exceed the surface tension and result in the ejection of a fiber jet from the apex of the cone which accelerates towards the grounded collector [51,52]. As the fiber jet accelerates towards the collector it undergoes chaotic whipping instability which increases the transit time and path length to the collector, allowing the solvent to evaporate leaving solid, thin fibers. Cheng et al. presented electrospinning as a novel processing method to generate functional nanomaterials with many applications ranging from wound healing and medical textiles, to production of oral-dispersible film SD and controlled delivery systems [27,53,54,55]. Electrospinning is a rapid solvent evaporation process well suited to amorphous materials as by this process the drug does not have time to form a crystal lattice within the nanofiber and remains distributed in its amorphous form at a molecular scale [56]. Additionally, the internal structure of the fibers themselves can be altered to be hollow or biphasic by using specific electrospinning approaches [57].

#### 2.1.1. Mono-Axial Electrospinning

Mono-axial electrospinning is the simplest and as a result of this the most common electrospinning experiment. The polymer (and API) in solution or suspension is dispensed through a single-bore blunt-end needle resulting in a monolithic product, with the API and polymer typically evenly and homogeneously blended throughout the fibers [58].

In Verreck et al.’s study, Itraconazole-HPMC SDs were generated by electrospinning and properties of the resultant nanofibres were compared relative to other SD techniques namely HME and solvent casting with regards their dissolution behavior [59]. Though the dissolution methods varied for the different presentations, several apparent trends were observed. PMs of itraconazole and HPMC generated little (<1–3%) drug release in any of the dissolution approaches assessed. Interestingly, electrospun ASDs samples resulted in complete in vitroitraconazole release over but however, the dissolution rate was slower compared to the cast thin films, melt extruded and milled powders [59]. The authors concluded that for this drug-polymer system, there could be potential as a controlled drug delivery system. Nagy et al. compared the drug dissolution behavior of spironolactone-soluplus SDs generated by electrospinning to those prepared by HME with key findings confirming the generation of ASDs with the electrospun nanofibers showing enhanced drug dissolution behavior compared to the HME SDs [60,61].

PVP-acetaminophen SDs prepared by electrospinning generating non-woven nanofibrous meshes were compared to traditional SD preparation processes namely freeze drying, vacuum drying, and heat drying with an advantage of the electrospinning process over the traditional processes being the unique microstructural characteristics of the electrospun nanofibres generated. Freeze dried samples and electrospun fibers did not show any evidence of phase separation phenomenon which was evident in heat-dried and vacuum-dried samples which led to visible (in scanning electron microscopy studies) microparticles on the sample surfaces. The in vitro dissolution tests illustrated that the electrospun SDs nanofibers released 93.8% acetaminophen in the first 2 min and the dissolution rates of acetaminophen from the different SDs had the following order: electrospun > vacuum-dried ≈ freeze-dried > heating-dried [62]. They also highlighted [62] the advantage of third generation SDs (where the state the carrier has surface activity or self-emulsifying properties) and the potential for these to be generated using mono-axial electrospinning. Third generation SDs of ferulic acid in composite nanofibers with three-dimensional continuous web structure were generated by electrospinning co-dissolving solutions of ferulic acid, PVP with the surfactant sodium dodecyl sulfate (SDS) and sucralose. In vitro dissolution and permeation tests showed that the nanofiber-based SDs rapidly released all of the ferulic acid within in 1 min and had a 13-fold greater permeation rate across sublingual mucosa compared to crude ferulic acid particles [63,64].

#### 2.1.2. Coaxial and Multi-Axial Electrospinning

Coaxial electrospinning (also known as co-electrospinning) involves the use of a two-needle spinneret, with one needle nested inside another in a concentric fashion. Fires generated tend to have greater versatility in terms of the range of materials used, therapeutic agents incorporated (ranging from small molecules to biological molecules), size (e.g., from 100 nm to 300 μm), and can offer complete drug encapsulation and higher drug stability compared to mono-axial fibers [58]. The use of coaxial and multi-axial electrospinning approaches has been applied in drug delivery with great success to generate biphasic [65], targeted release [66,67] and multifunctional materials [68] as well as SDs. Yang et al. [69] explored three types of electrospinning namely coaxial, modified coaxial, and tri-axial (characterized by the use of un-spinnable liquids as the sheath working fluids) electrospinning to generate nanofibrous ferulic acid-cellulose acetate ASDs depot systems [69]. In vitro dissolution tests revealed that the fibers were able to provide close to zero-order release over 36 h, with no initial burst release and minimal tailing-off. The release properties of the depot systems generated showed improved properties over the monolithic fibers, which exhibited a significant burst release and also considerable tailing-off at the end of the drug release studies [69]. Tri-axial electrospinning has also been successfully used to fabricate nanofibres of lecithin (known to be un-spinnable), diclofenac sodium with Eudragit S100 a methacrylic acid/methyl methacrylate copolymer (which only dissolves at pH > 7.0) as the carrier matrix for oral colon targeting [70].

#### 2.1.3. Others

Several other electrospinning systems exist which have been used to generate ASDs. The core-cut nozzle system where the exit pipe of the core nozzle is removed so that the core fluid can form an envelope inside the shell solution [71] has been shown to improve spinnability of two fluids by reducing jet instability. This system is also suitable for the production of multi-layered nanofibers where the drug is in the internal layer. Due to this, drug release is shown to start from the core then moving through to the external environment with applications as a controlled drug delivery system as the thickness of each layer can be tailored to vary the drug release [71]. Side-by-side electrospinning is a two-liquid process and the structure of the spinneret is propagated into the solid products creating Janus fibers with two different sides [72]. The different sides could hold two different polymers with two different drugs loaded resulting in concomitant drug release [73]. 

Unfortunately, in spite of the enormous research efforts both in academia and in industry very few ASDs products let alone nanofibrous ASDs have reached the market so far [74]. Works by Démuth et al. have successfully turned ASDs nanofibers into a viable nanofibrous ASDs solid dosage forms by compressing them into tablets with enhanced drug dissolution rates [75,76]. With promising work on-going in the field, there is the potential to have a commercially available nanofibrous ASD based dosage form.

## 3. Nanofibrous Micro/Nano-Crystalline Solid Dispersions

Due to the challenges associated with the stability of ASDs, there is a push to reconsider the crystalline state in the micron or nanoscale as an alternative method for SD production. It is well-known that the dissolution rate of smaller particles is greater due to the increased surface area in accordance with Noyes–Whitney equation and Nernst–Brunner theory [66,67,68]. Several investigations into drug particle size reduction [77,78,79] have shown that particle size reduction leads to increased drug solubility. It has also been demonstrated that a further increase in drug bioavailability is observed when the particle size of a drug is reduced from micrometer to nanometer [80].

Nanosuspensions are sub-micron colloidal dispersions of the drug, which are stabilized by surfactants [81]. This technology has shown great promise as a strategy to improve the dissolution rate of poorly soluble drugs by maintaining the drug in its preferred crystalline state of a size sufficiently small for pharmaceutical acceptability [82,83]. Major advantages of this technology are its general applicability to most drugs and its simplicity [80]. Solid micro/nano-crystalline dispersions have been generated using techniques such as film making [82,84], solvent casting method [32], spray drying [84,85], hot melt extrusion [25], and electrospinning [86].

## 4. Conclusions

In this review, we focused on the emerging trends in the generation of amorphous and micro/nano-crystalline solid dispersions using electrospinning to improve the dissolution rate and in turn the bioavailability of poorly water-soluble drugs. With electrospinning being a simple but versatile process, various studies have successfully generated mainly amorphous solid dispersions with few studies exploring the generation of crystalline solid dispersions. The scalability of electrospinning and its products for commercial drug delivery applications is a topic of great discussion and debate. The industrial upscaling of electrospinning and application of resultant fibers is discussed by Taylor et al. [86]. The electrospinning equipment market for laboratory research and industrial production is expected to grow significantly in the future due to the continuous development of new electrospinning technologies influenced by demand.
